# Genome-wide characterization of developmental stage- and tissue-specific transcription factors in wheat

**DOI:** 10.1186/s12864-015-1313-y

**Published:** 2015-02-25

**Authors:** Zhen-Yong Chen, Xiao-Jiang Guo, Zhong-Xu Chen, Wei-Ying Chen, Deng-Cai Liu, You-Liang Zheng, Ya-Xi Liu, Yu-Ming Wei, Ji-Rui Wang

**Affiliations:** Triticeae Research Institute, Sichuan Agricultural University, Wenjiang, Chengdu, 611130 China; College of Life Science, China West Normal University, Nanchong, 637009 China; Ministry of Education Key Laboratory for Crop Genetic Resources and Improvement in Southwest China, Sichuan Agricultural University, Yaan, Sichuan 625014 China

**Keywords:** Transcription factor, *Triticum aestivum*, WheatTFDB, Developmental stage, Tissue

## Abstract

**Background:**

Wheat (*Triticum aestivum*) is one of the most important cereal crops, providing food for humans and feed for other animals. However, its productivity is challenged by various biotic and abiotic stresses such as fungal diseases, insects, drought, salinity, and cold. Transcription factors (TFs) regulate gene expression in different tissues and at various developmental stages in plants and animals, and they can be identified and classified into families according to their structural and specialized DNA-binding domains (DBDs). Transcription factors are important regulatory components of the genome, and are the main targets for engineering stress tolerance.

**Results:**

In total, 2407 putative TFs were identified from wheat expressed sequence tags, and then classified into 63 families by using Hmm searches against hidden Markov model (HMM) profiles. In this study, 2407 TFs represented approximately 2.22% of all genes in the wheat genome, a smaller proportion than those reported for other cereals in PlantTFDB V3.0 (3.33%–5.86%) and PlnTFDB (4.30%–6.46%). We assembled information from the various databases for individual TFs, including annotations and details of their developmental stage- and tissue-specific expression patterns. Based on this information, we identified 1257 developmental stage-specific TFs and 1104 tissue-specific TFs, accounting for 52.22% and 45.87% of the 2407 wheat TFs, respectively. We identified 338, 269, 262, 175, 49, and 18 tissue-specific TFs in the flower, seed, root, leaf, stem, and crown, respectively. There were 100, 6, 342, 141, 390, and 278 TFs specifically expressed at the dormant seed, germinating seed, reproductive, ripening, seedling, and vegetative stages, respectively. We constructed a comprehensive database of wheat TFs, designated as WheatTFDB (http://xms.sicau.edu.cn/wheatTFDB/).

**Conclusions:**

Approximately 2.22% (2407 genes) of all genes in the wheat genome were identified as TFs, and were clustered into 63 TF families. We identified 1257 developmental stage-specific TFs and 1104 tissue-specific TFs, based on information about their developmental- and tissue-specific expression patterns obtained from publicly available gene expression databases. The 2407 wheat TFs and their annotations are summarized in our database, WheatTFDB. These data will be useful identifying target TFs involved in the stress response at a particular stage of development.

**Electronic supplementary material:**

The online version of this article (doi:10.1186/s12864-015-1313-y) contains supplementary material, which is available to authorized users.

## Background

Common wheat (*Triticum aestivum* L.) is the most important and widespread cultivated food crop in the world. The approximate global output of wheat was 711.42 million tons in 2013 [1]. Wheat is an essential source of protein, vitamins, and minerals for humans. Therefore, many studies, including genetic, genomic, and proteomic studies, have concentrated on improving wheat productivity. The genome of hexaploid wheat contains 16,000 Mb of DNA originating from the natural hybridization of three genomes; A, B, and D [2]. It originated from the spontaneous hybridization of tetraploid emmer wheat (AABB, *Triticum dicoccoides*) with diploid goat grass (DD, *Aegilops tauschii*), while the *Triticum urartu* (AA) and BB genomes from an unknown species (close to modern *Aegilops speltoides*) naturally combined to form tetraploid emmer wheat [3]. The A, B, and D genomes show extensive and high conservation [3]. Although the genome survey sequences of wheat [3], *Triticum urartu* [4], and *Aegilops tauschii* [5] have been published, the reference sequences of the wheat genome have not been completed because of its complex polyploidy and homology. Despite the lack of reference sequence information, major efforts are underway to increase the output of wheat by genetically analyzing its traits and increasing the genetic diversity of the breeding materials. At the same time, studies on the wheat proteome are underway, including research on transcription factors (TFs) [6-8].

Transcription factors are proteins that are expressed in different organs, and at different developmental stages, in plants and animals. These proteins regulate the gene expression level by binding to *cis*-regulatory elements in the promoters of target genes to control various biological processes such as growth, cell division, and responses to the environment or stress [9]. The sequence region of TFs that binds to the target genes is the DNA-binding domain (DBD). Usually, TFs can be grouped into families according to the structural features of conserved DBDs. Some TF databases group TFs based on these families. A genome-wide comparative analysis of TFs in *Arabidopsis thaliana* and other eukaryotic genomes was completed by Riechmann *et al.*, revealing 1533 TFs in the *A. thaliana* genome [10]. Subsequently, several plant TF databases have been established and are continuously updated; e.g., the RIKEN *Arabidopsis* Transcription Factor Database (RARTF) [11], the Plant Transcription Factor Database (PlnTFDB) [12], and the Plant Transcription Factor Database (PlantTFDB) [13,14]. The latest version of PlnTFDB (V3.0) contains TFs from 19 plant species, and includes TF databases of rice, sorghum, and maize [15]. PlantTFDB V3.0 provides TF databases for 83 plant species, including African rice, barley, and wheat [14].

In 2009, Romeuf *et al.* constructed the first publicly available wheat TF database, the Database of Wheat Transcription Factors (wDBTF), by BlastX alignment against rice TF sequences in the Database of Rice Transcription Factors (DRTF) [16]. wDBTF contains 7112 putative wheat TF sequences belonging to 84 subfamilies. In the database, expression data and tissue source information are provided for individual sequences, and this information can be used to identify tissue-specific wheat TFs. However, the data in wDBTF have not been updated since 2009. The subsequently published wheat TF set in PlantTFDB V2.0 included 746 TF sequences [17]. PlantTFDB V2.0 listed additional information for each TF, including functional information such as the domain features, gene ontology (GO) terms, and phylogenetic tree of the TF family. This database was updated to version 3.0 in 2013 [14]. At present, PlantTFDB V3.0 includes information for 1940 wheat TFs, which provides users with data to study their functions. However, the wheat TF sequences in PlantTFDB V3.0 are not accompanied by data on tissue and developmental expression patterns, which is useful for studying the function and evolution of TFs [18-20]. Thus, it is necessary to list TFs with information about their developmental- and tissue-specific expression patterns.

The objectives of this research were as follows: (i) to construct a wheat TF database that includes developmental- and tissue-specific expression information for individual TF sequences; and (ii) to identify TFs that are specific to particular developmental stages and tissues.

## Results

In total, 2407 wheat TFs obtained by Hmm searches were classified into 63 families. Among them, 19 families contained more than 35 TFs, which was the average number of TFs per family. There were 1016 TFs predicted from singletons, accounting for 42.21% of the 2407 TFs (Additional file 1: Table S2). The number of TFs in each TF family in our database, WheatTFDB, is shown in Table 1. The myeloblastosis (MYB) superfamily, including the MYB and MYB-related families, was the largest family, with 127 MYB and 128 MYB-related members. There were more than 100 members of the following families: ethylene responsive factor (ERF); B3; basic helix-loop-helix (bHLH); basic region/leucine zipper motif (bZIP); NAM, ATAF, and CUC (NAC); nuclear transcription factor Y, gamma (NF-YC); and WRKY TF. The ARR-B, SRS, and ULT families each had a single TF. There were no TFs in the HB-PHD, homeodomain leucine zipper (HD-ZIP), hairy-related transcription-factor-like (HRT-like), LEAFY (LFY), nuclear transcription factor, X-box binding 1 (NF-X1), and NOZZLE/SPOROCYTELESS (NZZ/SPL) families; therefore, these six families were removed from WheatTFDB.

**Table 1:** **Numbers of every transcription factor family in WheatTFDB**

**Family**	**TF number**	**Family**	**TF number**	**Family**	**TF number**
Alfin-like	21	FHA	19	NF-YB	29
AP2	9	G2-like	32	NF-YC	102
ARF	10	GATA	22	Nin-like	10
ARR-B	1	GeBP	6	NZZ/SPL	0
B3	106	GRAS	81	OFP	17
BBR-BPC	5	GRF	3	PLATZ	17
BES1	4	HB-other	83	RAV	4
bHLH	140	HB-PHD	0	S1Fa-like	11
BSD	13	HD-ZIP	0	SBP	12
bZIP	110	HRT-like	0	Sigma70-like	6
C2H2	48	HSF	42	SRS	1
C3H	58	LBD(AS2/LOB)	25	TALE	8
CAMTA	4	LFY	0	TAZ	8
CO-like	4	LIM	24	TCP	10
CPP	5	LSD	6	Tify	75
CSD	38	MIKC	50	Trihelix	28
DBB	33	mTERF	77	TUB	34
DBP	2	M-type	41	ULT	1
Dof	32	MYB	127	VOZ	6
E2F/DP	5	MYB-related	128	Whirly	7
EIL	14	NAC	193	WRKY	135
ERF	217	NF-X1	0	YABBY	10
FAR1	14	NF-YA	11	ZF-HD	13

WheatTFDB: wheat transcription factor database.

We surveyed the original developmental stage information of the 2407 TFs. The details of the TFs expressed at different developmental stages are listed in Additional file 2: Table S3. This table contains several columns: family, TF ID, and presence at the dormant seed, germinating seed, reproductive, ripening, seedling, vegetative, and “unclear” developmental stages. The “family” column represents the 63 TF families mentioned above. The “TF ID” column shows the accession numbers of the 2407 TF sequences in WheatTFDB. The presence of the TF at a given developmental stage is indicated by the value “1” in the relevant column (e.g., dormant seed, germinating seed, or reproductive stage). Based on this definition, we identified 20 TFs at six developmental stages that regulate biological processes. These TFs included TaTF00003 in the Alfin-like family; TaTF00347 in the bZIP family; TaTF01944, TaTF01916, and TaTF01988 in the NF-YC family; TaTF00664 and TaTF00679 in the ERF family; and TaTF02196 in the trihelix family (Additional file 2: Table S3). Of the 2407 TFs, we initially identified 1326 putative developmental stage-specific TFs. The developmental stage information of these 1326 putative specific TFs was validated by BLAST searches in the NCBI EST database (Additional file 3: Table S5). We obtained 11,492 subject sequences in the NCBI EST database with the 1326 putative TF sequences (Additional file 3: Table S5). However, only 1120 of the subject sequences were matched sequences (Additional file 4: Table S7). We validated and updated the developmental stage information of the 1326 putative specific sequences in Additional file 2: Table S3 (shown with a red background) according to the BLAST results listed in Additional file 4: Table S7. Then, we obtained developmental stage information for the filtered-out TFs. As identified by Hmm searches, 762 TFs were identified from the filtered-out sequences. Each redundant TF sequence was clustered and matched to a TF sequence in the identified 2407 TF set. Then, we retrieved information on the developmental stage of the 762 filtered-out TFs. This resulted in new developmental stage information for 282 of the filtered-out sequences. This developmental stage data was added to Additional file 2: Table S3 (highlighted with a green background).

When the value in the ninth column in Additional file 2: Table S3 was 1, the TF was a stage-specific TF. We obtained 1257 developmental stage-specific sequences in this study (Table 2). The six large TF families (ERF, NAC, WRKY, bHLH, MYB, and B3) contained 516 TFs, accounting for 41.05% of the 1257 TFs. The ARR-B, CO-like, albumin D-binding protein (DBP), and ULT families were unspecific wheat TF families, because these families did not include any specific TFs.

**Table 2:** **Specific transcription factor numbers in wheat identified at different developmental stages**

**Family**	**Dormant seed**	**Germinating seed**	**Reproductive**	**Ripening**	**Seedling**	**Vegetative**	**Sum of specific TF in each family**	**Unclear**
Alfin-like	2	0	2	0	1	1	6	2
AP2	0	0	2	0	0	1	3	0
ARF	0	0	1	0	0	0	1	0
ARR-B	0	0	0	0	0	0	0	0
B3	4	0	36	5	13	6	64	7
BBR-BPC	0	0	0	1	0	0	1	1
BES1	2	0	0	0	0	0	2	1
bHLH	4	0	22	5	29	15	75	20
BSD	0	0	1	1	1	0	3	2
bZIP	7	0	7	2	23	7	46	9
C2H2	0	0	11	2	4	4	21	8
C3H	2	0	13	5	6	5	31	1
CAMTA	0	0	0	0	2	0	2	1
CO-like	0	0	0	0	0	0	0	0
CPP	0	0	2	0	0	0	2	0
CSD	2	1	2	3	11	3	22	7
DBB	0	0	2	1	3	14	20	2
DBP	0	0	0	0	0	0	0	0
Dof	3	0	4	3	4	3	17	4
E2F/DP	1	0	1	0	1	0	3	0
EIL	1	0	2	0	2	2	7	0
ERF	8	0	26	17	35	44	130	25
FAR1	2	0	4	1	1	0	8	3
FHA	0	0	3	0	2	2	7	1
G2-like	1	0	3	0	7	0	11	6
GATA	2	0	3	0	1	1	7	2
GeBP	0	0	2	0	1	0	3	0
GRAS	4	0	15	10	19	2	50	6
GRF	0	0	1	0	0	0	1	1
HB-other	5	2	12	9	9	9	46	9
HSF	2	0	6	2	9	3	22	1
LBD(AS2/LOB)	2	0	4	3	6	3	18	4
LIM	2	0	4	1	6	2	15	2
LSD	0	0	0	0	3	0	3	1
MIKC	0	0	14	0	2	2	18	3
mTERF	5	0	20	11	8	3	47	13
M-type	3	0	9	5	11	2	30	3
MYB	2	0	18	8	15	24	67	14
MYB-related	8	0	8	6	32	9	63	12
NAC	1	0	16	15	33	40	105	16
NF-YA	1	0	1	0	0	1	3	1
NF-YB	1	0	6	3	2	3	15	3
NF-YC	3	2	7	5	10	17	44	10
Nin-like	1	0	1	0	4	0	6	0
OFP	3	0	2	1	5	2	13	2
PLATZ	1	0	1	3	2	3	10	1
RAV	0	0	0	0	2	1	3	1
S1Fa-like	0	0	1	2	4	0	7	1
SBP	0	0	6	0	1	0	7	3
sigma70-like	0	0	0	0	2	0	2	0
SRS	0	0	0	0	0	1	1	0
TALE	0	0	2	0	0	1	3	0
TAZ	2	0	1	0	3	0	6	0
TCP	0	0	2	0	0	1	3	0
Tify	0	0	12	1	10	13	36	4
Trihelix	3	0	4	3	3	2	15	1
TUB	0	1	1	1	9	2	14	3
ULT	0	0	0	0	0	0	0	0
VOZ	0	0	0	0	2	0	2	1
Whirly	1	0	0	0	2	0	3	2
WRKY	7	0	13	4	28	23	75	11
YABBY	0	0	2	2	0	0	4	1
ZF-HD	2	0	4	0	1	1	8	2
Sum of specific TFs at different stage	100	6	342	141	390	278	1257	234
Percentage of specific TFs at different stage	4.15%	0.25%	14.21%	5.86%	16.20%	11.55%	52.22%	9.72%

Sum of specific TF in each family were the sum of TF numbers from dormant seed, germinating seed, reproductive, ripening, seedling and vegetative.

Percentage of specific TFs at different stage was divided specific TF numbers at different stage by 2407.

We identified 100 specific TF sequences at the dormant seed stage. The MYB superfamily, ERF, bZIP, WRKY, and mitochondrial transcription termination factor (mTERF) families accounted for 37 (37.0%) of the 100 specific TFs at the dormant seed stage. At the germinating seed stage, there were six stage-specific TFs belonging to four families; the NF-YC, HB-other, Tubby (TUB), and cold shock domain (CSD) families had 2, 2, 1, and 1 sequence(s), respectively. We found 342 specific sequences in 50 families at the reproductive stage; the families with the most abundant transcripts were the B3, ERF, bHLH, mTERF, and MYB families, which together accounted for 122 (35.67%) of the 342 TFs. In ripening wheat, there were 141 stage-specific sequences in 33 families, and the ERF, NAC, mTERF, GRAS, HB-other, and MYB families accounted for 53.90% of the 141 sequences. At the seedling stage, 390 TFs belonging to 49 families were stage-specific TFs. Of these, 195 TFs (50%) belonged to the six most abundant families; the MYB superfamily, and the ERF, NAC, WRKY, bHLH, and bZIP families. At the vegetative stage, 278 TFs belonging to 38 families were stage-specific, and the ERF, NAC, MYB, WRKY, NF-YC, and double B-box (DBB) families accounted for 56.47% of the 278 stage-specific sequences. When the sum of “each TF present at dormant seed, germinating seed, reproductive, ripening, seedling, and vegetative stage” was equal to zero, and the value in the “unclear developmental stage” column was 1, the developmental stage of the TF was unclear. There were 234 unclear TFs among the 2407 TFs. The developmental stage information for these TFs should be confirmed in future research.

**Figure 1:**
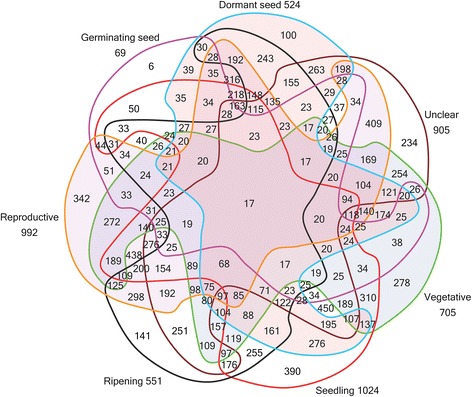
**Distribution of wheat transcription factors at different developmental stages.**

Distribution information for the individual TFs at different developmental stages is shown in Figure 1. To provide further explanation for the information in Figure 1, Additional file 5: Figure S1 shows the interpretation of Grünbaum’s seven-set Venn diagram [21]. The number of stage-specific TFs at the dormant seed, germinating seed, ripening, reproductive, seedling, vegetative, and unclear stages was 524, 69, 992, 551, 1024, 705, and 905, respectively. Only 20 TFs were expressed at all six developmental stages (Figure 1).

The tissue source information for the 2407 TFs is summarized in Additional file 6: Table S4. When the value in the ninth column was 1, the TF was designated as a tissue-specific TF. We initially distinguished 1233 putative tissue-specific TFs among the 2407 wheat TFs (Additional file 6: Table S4). Then, the tissue information of the 1233 putative specific TFs was validated by BLAST searches in the NCBI wheat EST database. We matched 10,511 subject sequences with the 1233 original ESTs that were putative tissue-specific sequences (Additional file 7: Table S6). There were 1151 matched sequences among 10,511 subject sequences (Additional file 8: Table S8). Among these 1151 sequences, 88 sequences had different tissue information from that listed with the 1233 putative tissue-specific TFs. We updated the tissue information for these 88 specific TFs in Additional file 6: Table S4 (highlighted with a red background). We also analyzed the tissue information for the 762 filtered-out TFs. In total, 277 new tissue information items for the filtered sequences were added to Additional file 6: Table S4 (highlighted with a green background). Finally, 1104 tissue-specific sequences were obtained.

**Figure 2:**
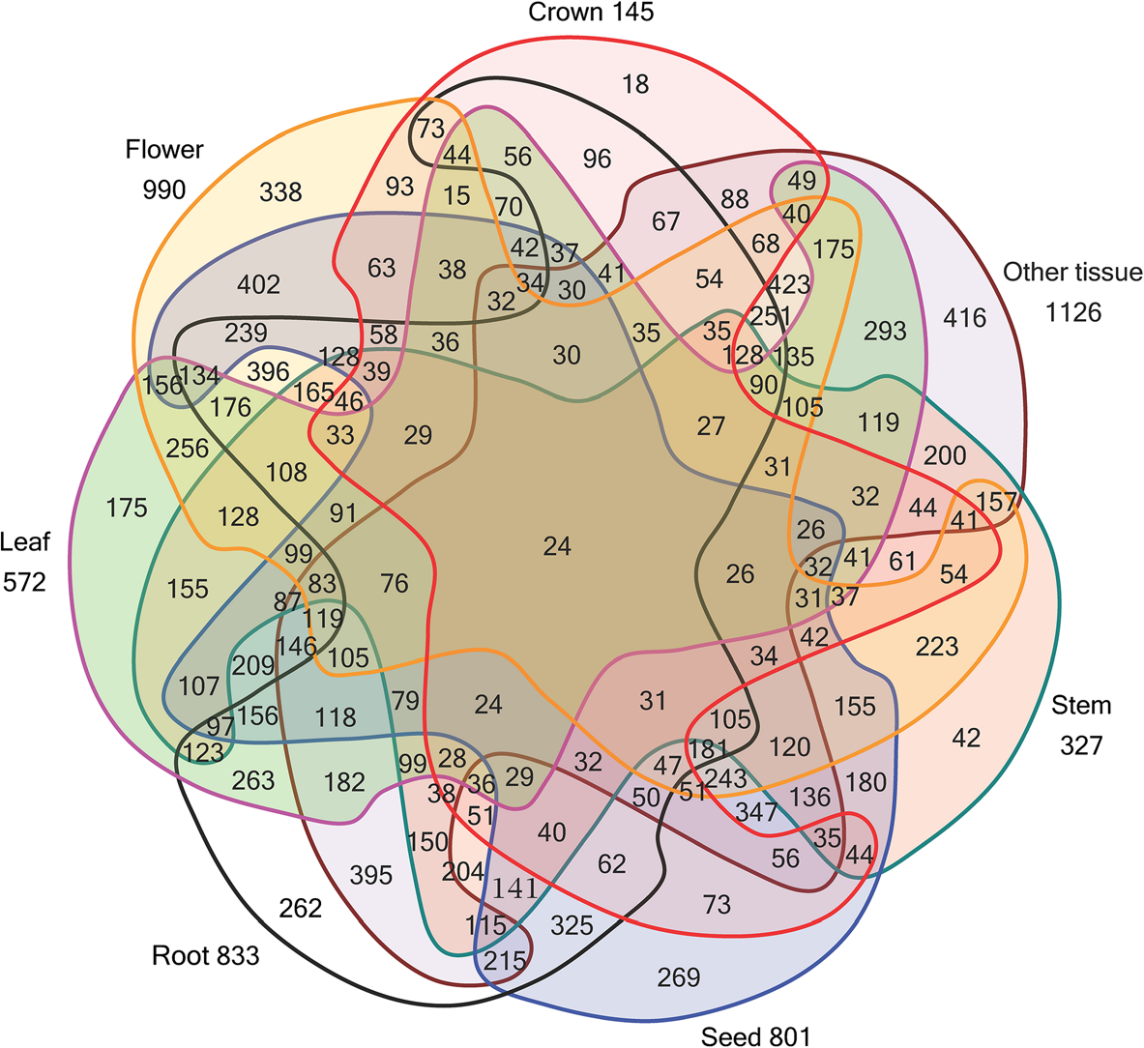
**Distribution of wheat transcription factors from different tissues.**

Table 3 shows the number of wheat tissue-specific TFs in the 63 families. The first six subfamilies (ERF, NAC, bHLH, B3, MYB, and WRKY) contained 434 (39.31%) of the 1104 TFs, with each family containing more than 60 TFs. The ARR-B, CO-like, DBP, and ULT families were unspecific TF families, and contained no tissue-specific TFs. As shown in Table 3, 18 sequences belonging to 14 families were specific TFs in the crown. When we analyzed the families of the 21 crown-specific TFs, the three largest tissue-specific TF families in crown were the bHLH, WRKY, and mTERF families, containing 3, 2, and 2 sequences, respectively. In the wheat flower, 338 TFs belonging to 46 families were tissue-specific TFs. Of these, 152 (44.97%) belonged to the MYB superfamily and the B3, ERF, mTERF, NAC, and WRKY families. In total, 175 specific TFs belonging to 37 families were identified in the wheat leaf. The NAC, ERF, MYB superfamily, WRKY, Tify, and bHLH families were the six most abundant TF families, accounting for 54.29% of the 175 leaf-specific TFs. There were 266 sequences belonging to 45 families that were specifically expressed in the wheat root. Among them, 121 (46.18%) were from the ERF, bHLH, WRKY, and bZIP families and the MYB superfamily. Forty-two sequences in 21 families were specifically expressed in the wheat stem. The larger families, the MYB superfamily and the mTERF and bHLH families, accounted for 16 (38.10%) of the total stem-specific wheat TFs. There were 269 TFs in 47 families that were exclusively expressed in the seed. The NAC, ERF, and HB-other families and the MYB superfamily were the four largest families, accounting for 34.57% of the 269 TFs. Grünbaum’s seven-set Venn diagram shows the distribution of TFs among the crown, flower, leaf, root, seed, stem, and other wheat tissues (Figure 2). Additional file 5: Figure S1 facilitates the interpretation of Figure 2 [21]. The number of wheat TFs identified in the crown, flower, leaf, root, seed, stem, and other tissues was 145, 990, 572, 833, 801, 327, and 1126, respectively (Figure 2). Twenty-nine TFs were expressed in the crown, flower, leaf, root, seed, and stem (Additional file 6: Table S4), including TaTF00036 in the ARF family; TaTF00209 in the bHLH family; and TaTF02194 in the trihelix family. When the value of “sum of each TF appears at crown, flower, leaf, root, seed, and stem” was equal to zero and the value in the column for “other tissue” was 1, the tissue type of the TF was designated as “other tissue”. Of the 1104 TFs in wheat, 416 were in the “other tissue” group (Figure 2, Table 3).

**Table 3:** **Numbers of tissue specific transcription factor in wheat**

**Family**	**Crown**	**Flower**	**Leaf**	**Root**	**Seed**	**Stem**	**Sum of specific TF in each family**	**Other tissue**
Alfin-like	0	3	2	1	3	0	9	1
AP2	0	0	0	1	1	0	2	0
ARF	0	3	0	0	0	0	3	0
ARR-B	0	0	0	0	0	0	0	0
B3	1	40	5	7	11	0	64	12
BBR-BPC	0	1	0	0	1	1	3	0
BES1	0	0	0	2	0	0	2	0
bHLH	3	18	13	25	9	5	73	23
BSD	0	0	0	2	1	0	3	1
bZIP	0	7	3	17	11	3	41	13
C2H2	0	10	1	8	4	0	23	8
C3H	0	10	1	1	8	1	21	9
CAMTA	0	0	0	1	0	1	2	1
CO-like	0	0	0	0	0	0	0	0
CPP	0	3	0	0	1	0	4	0
CSD	0	3	3	11	5	0	22	6
DBB	0	1	7	2	1	0	11	13
DBP	0	0	0	0	0	0	0	0
Dof	0	4	4	2	7	1	18	4
E2F/DP	0	1	1	0	1	0	3	1
EIL	0	1	2	0	2	0	5	2
ERF	1	23	12	24	23	1	84	64
FAR1	0	5	0	1	2	1	9	3
FHA	0	4	1	1	0	0	6	2
G2-like	1	3	3	3	2	3	15	4
GATA	0	2	0	2	5	0	9	2
GeBP	0	2	0	0	0	0	2	0
GRAS	1	9	10	6	14	0	40	7
GRF	0	1	0	0	0	0	1	1
HB-other	0	10	4	6	19	2	41	15
HSF	0	8	2	2	7	0	19	11
LBD(AS2/LOB)	1	5	1	5	4	0	16	5
LIM	0	4	2	5	2	0	13	3
LSD	0	0	2	0	0	0	2	2
MIKC	1	12	0	2	2	1	18	5
mTERF	2	21	2	7	12	5	49	8
M-type	0	9	3	1	5	1	19	9
MYB	0	23	5	17	13	4	62	24
MYB-related	0	8	16	17	11	2	54	20
NAC	1	15	29	15	27	2	89	51
NF-YA	0	0	0	1	1	0	2	1
NF-YB	0	3	2	2	6	1	14	3
NF-YC	1	10	3	11	13	0	38	19
Nin-like	0	0	0	3	1	0	4	1
OFP	0	2	2	5	3	0	12	2
PLATZ	0	0	0	3	4	0	7	3
RAV	0	0	0	1	0	0	1	3
S1Fa-like	0	1	0	3	1	0	5	0
SBP	1	4	0	0	1	1	7	2
sigma70-like	0	0	1	0	0	0	1	0
SRS	0	0	0	1	0	0	1	0
TALE	0	2	0	1	0	0	3	0
TAZ	0	1	1	1	1	0	4	0
TCP	0	1	0	0	1	0	2	0
Tify	1	13	10	9	3	2	38	14
Trihelix	0	4	3	1	7	0	15	2
TUB	0	3	3	4	1	1	12	6
ULT	0	0	0	0	0	0	0	0
VOZ	0	0	2	1	0	0	3	0
Whirly	1	0	1	0	1	0	3	0
WRKY	2	19	10	21	7	3	62	27
YABBY	0	3	0	0	2	0	5	1
ZF-HD	0	3	3	0	2	0	8	2
Sum of specific TFs in different tissue	18	338	175	262	269	42	1104	416
Percentage of tissue specific TFs	0.75%	14.04%	7.27%	10.88%	11.18%	1.74%	45.87%	17.28%

“Specify TF number of every family” is the sum of the TF numbers from the crown, flower, leaf, root, seed, and stem.

Percentage of tissue specific TFs was obtained by dividing the number of specific TFs from different tissues by 2407.

The redundancies of TFs among wDBTF, PlantTFDB, and WheatTFDB are shown in Table 4. WheatTFDB did not have any redundant TFs above a similarity threshold of 95%, and PlantTFDB did not have any similar sequences at the 100% threshold. wDBTF showed various degrees of redundancy at the four similarity thresholds. There were 510 redundant TFs at a similarity threshold of 100%. At each similarity threshold, more redundant sequences were identified in wDBTF than in PlantTFDB and WheatTFDB. The similarities in the three wheat TF databases were evaluated using the cdhit program. In all three databases, the number of similar sequences decreased as the similarity threshold increased. At the four similarity thresholds, the number of similar TFs was higher in wDBTF than in WheatTFDB. There were 1659 redundant sequences among the three TF databases at a similarity threshold of 100% (Table 4). The 1659 redundant TFs consisted of 377 sequences from WheatTFDB, 122 from PlantTFDB, and 1160 TFs from wDBTF, which were clustered as similar sequences at a similarity threshold of 100%.

**Table 4:** **Comparison of the matches of wheat transcription factors database at four similarity thresholds**

**Similarity**	**WheatTFDB-WheatTFDB**	**wDBTF-wDBTF**	**PlantTFDB-PlantTFDB**	**wDBTF-WheatTFDB**	**wDBTF-PlantTFDB**	**WheatTFDB-PlantTFDB**	**WheatTFDB-wDBTF-PlantTFDB**
85%	639	2579	328	4285	3918	1882	5832
90%	381	2116	190	3607	3281	1494	5001
95%	0	1534	21	2691	2422	873	3810
100%	0	510	0	1212	829	254	1659

wDBTF-WheatTFDB represented the similarity between wDBTF and WheatTFDB;

WheatTFDB-WheatTFDB represented the similarity in WheatTFDB itself;

WheatTFDB-wDBTF-PlantTFDB represented the similarity among WheatTFDB, wDBTF, and PlantTFDB.

We compared the percentages of TFs in genomes of various cereals among the different databases (Table 5). The percentage of TFs in cereal genomes was 3.33%–5.86% in PlantTFDB V3.0 and 4.30%–6.46% in PlnTFDB V3.0. Both the number of TFs and their percentage in the wheat genome were higher in wDBTF (7112 and 6.55%, respectively) than in PlantTFDB (1940 and 3.46%, respectively) and WheatTFDB (2407 and 2.22%, respectively).

**Table 5:** **Transcription factors numbers and percentages of grasses in different database**

**Species**	**PlantTFDB V3.0** ^**a**^	**PlnTFDB V3.0** ^**b**^	**wDBTF** ^**c**^	**WheatTFDB** ^**d**^
*Brachypodium distachyon*	1557(5.86%)	–	–	–
*Oryza sativa subsp. Indica*	1891(4.64%)	2393(4.82%)	–	–
*Oryza sativa subsp. Japonica*	1859(3.33%)	2722(4.30%)	–	–
*Sorghum bicolor*	1826(5.53%)	2231(6.25%)	–	–
*Hordeum vulgare*	1198(4.95%)	–	–	–
*Zea mays*	2231(5.73%)	3608(6.46%)	–	–
*Aegilops tauschii*	1439(4.25%)	–	–	–
*Triticum urartu*	888(3.67%)	–	–	–
*Triticum aestivum*	1940(3.46%)	–	7112(6.55%)	2407(2.22%)

^a^Plant Transcription Factor Database (http://planttfdb.cbi.pku.edu.cn).

^b^Plant Transcription Factor Database (http://plntfdb.bio.uni-potsdam.de).

^c^Database of Wheat Transcription Factor (http://wwwappli.nantes.inra.fr:8180/wDBFT/).

^d^Wheat transcription factor database (http://xms.sicau.edu.cn/wheatTFDB/).

In our new wheat TF database, WheatTFDB, we have provided functional information for each individual TF, including the protein domain identification and GO term assignment, which were obtained for the 2407 identified TFs using InterProScan V5.3–46.0. These annotations have been integrated into the page for each TFs in WheatTFDB. Additional file 9: Table S9 shows the genomic sequence information for the TFs, including their ID and chromosomal location. Some TFs were mapped to several sites on different chromosomes because of similarity among the genomes of wheat and duplication of genes during evolution (Additional file 9: Table S9).

**Figure 3:**
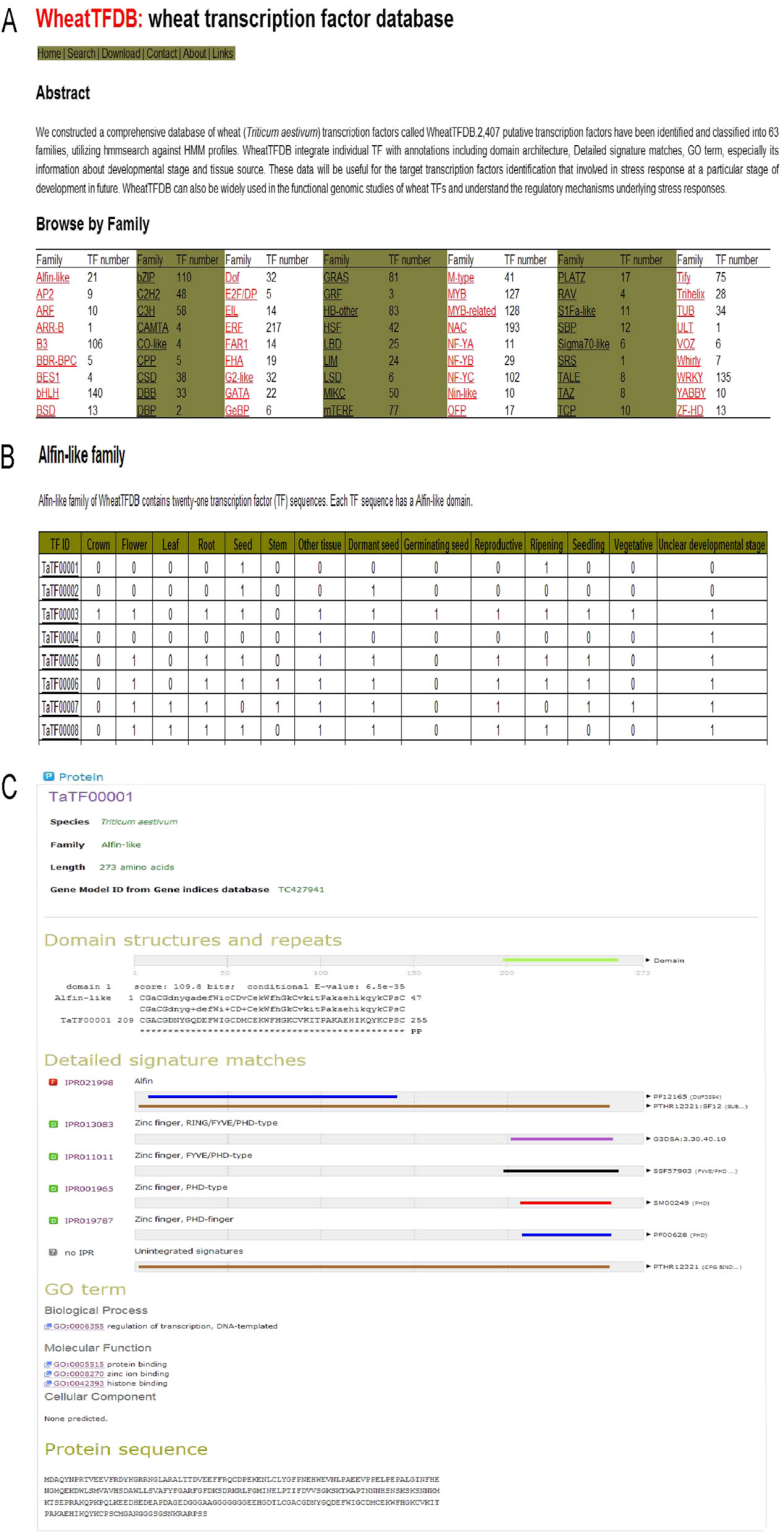
**The web interface of WheatTFDB.** The home page for WheatTFDB shows the list of 63 wheat TF families **(A)**. A typical page for a TF family displays the information of tissue resource and developmental stages of each TF **(B)**. The annotation information of individual TFs mainly contains four categories: domain structures and repeats, detailed signature matches in intrerPro V46.0, Gene ontology term and sequence **(C)**.

We constructed a wheat transcription factor database, WheatTFDB (http://xms.sicau.edu.cn/wheatTFDB/), based on the 2407 identified TFs. The TFs in WheatTFDB are grouped into 63 families, as shown on the WheatTFDB home page (Figure 3A). The TF family names listed on the home page have been hyperlinked to individual TF family pages. The individual TF family pages show information about the tissue sources and developmental stages of each TF (Figure 3B), and each TF is linked to its annotation page. The annotation page of each TF integrates information on domain structure, GO annotation, protein features, and sequence (Figure 3C). The additional files and TF sequences mentioned above have been deposited on the Download page of WheatTFDB (http://xms.sicau.edu.cn/wheatTFDB/Download.htm).

## Discussion

The wDBTF and wheat TF datasets in PlantTFDB V3.0 provide useful information for studying the functions of TF sequences. To identify the tissue- and developmental stage-specific TFs, we constructed a new wheat TF database, WheatTFDB. In wDBTF, wheat TFs were identified based on their DNA-binding motifs. Twelve DNA-binding with one finger (DOF) genes were confirmed to be involved in wheat grain development [16]. The putative TFs belonging to the other 83 subfamilies still need to be verified experimentally. The accuracy of wheat TFs in PlantTFDB V3.0 was tested using the methods described by Iida *et al.* [11] and Riano-Pachon *et al.* [12]. The results showed that the wheat TFs had acceptable accuracy [13]. The HMM profiles used in PlnTFDB and PlantTFDB have been verified, and have been used widely to identify TFs in other plants, for example rice, maize, and sorghum [12-15,17]. In this study, we used the same strategy to identify wheat TFs for WheatTFDB as that used for PlnTFDB and PlantTFDB; therefore, the TF families in WheatTFDB have acceptable accuracy, consistent with that of PlnTFDB and PlantTFDB.

We analyzed the redundancies and similarities within and among the three wheat TF databases (Table 4). The redundancy in wDBTF is much higher than that in WheatTFDB and PlantTFDB, as indicated by the higher number of redundant sequences in wDBTF at each threshold (Table 4). The similarities at each threshold indicate that wDBTF is most similar to WheatTFDB, and least similar to PlantTFDB, among the three databases. The three wheat TF databases provide 9800 non-redundant TFs at a similarity of 100%. This value is the sum of the 7112, 2407, and 1940 TFs in each database, after subtracting the 1659 redundant TFs. The 9800 non-redundant TFs consist of 2030 TFs from WheatTFDDB, 1818 TFs from PlantTFDB, and 5952 TFs from wDBTF. There are 2030 TFs in WheatTFDB that differ from those from PlantTFDB and wDBTF (i.e., 2407 TFs minus the 377 redundant TFs in WheatTFDB). Therefore, 2030 new TFs have been added to WheatTFDB, compared with those published in PlantTFDB and wDBTF. There are 1818 TFs in PlantTFDB and 5952 TFs in wDBTF that are not listed in WheatTFDB.

We calculated the percentages of TFs in the wheat genome in WheatTFDB and wDBTF, and compared these values with those in other cereals (Table 5). There are 108,569 protein-coding genes in wheat, close to the number predicted by Paux and Rachel *et al*. [3,16,22]. When the number of TFs was divided by 108,569 genes (Table 4), the percentage of TFs was higher in wDBTF than in WheatTFDB and PlantTFDB V3.0 (Table 5), because wDBTF has the highest number of TFs among the three wheat TF databases. In total, the three wheat TF databases predict 9800 TFs, accounting for 9.03% of the 108,569 wheat protein-coding genes. This percentage is higher than that expected for plants (5%–7%) [16]. This result indicates that the three wheat TF databases contain redundant sequences, especially wDBTF (Table 4). Thus, further experimental evidence is needed to confirm whether a predicted TF sequence is a TF or not.

Transcription factors have been shown to regulate gene expression, and are often expressed in specific tissues or at during specific developmental stages [18,23-25]. Some research on wheat TFs has focused on the function and/or evolution of several members of a particular TF family under various stress conditions [19,20,23,26-28]. There have also been studies on the molecular mechanisms of the responses of specific TFs to various abiotic or biotic conditions; such information may be used to improve the yield and quality of wheat through molecular breeding [5,24,26]. However, the systematic identification of wheat specific TFs in all of the TF families had not been performed. In this study, we identified 1257 TFs specific to different developmental stages and 1104 tissue-specific TFs, accounting for 52.22% and 45.87% of the 2407 wheat TFs, respectively. These results may facilitate studies on transcription regulation and on the evolution of specific wheat TFs.

A family-level analysis of 2407 wheat TFs showed that the MYB superfamily and the ERF and NAC families are the three largest TF families in common wheat. These three TF families accounted for 27.96% of the 2407 wheat TFs analyzed. Members of these families are abundant in *Arabidopsis*, rice, and maize [14,29,30]. The MYB superfamily is the largest group of TFs in wheat and maize [30]. The members of the MYB superfamily have been well studied in plants. Many studies have suggested that TFs in the MYB superfamily play central roles in the response to abiotic stresses and in developmental processes. Katiyar *et al*. identified 142 MYB genes that were expressed in the seedlings of rice, 92 of which were differentially regulated under drought stress. A comparison of the chromosomal distribution, tandem repeats, and phylogenetic relationships of MYB family genes in rice and *Arabidopsis* revealed their evolution via duplication [31]. In 2012, Zhang *et al*. studied 60 MYB genes isolated from the roots, stems, leaves, anthers, and pistils of Chinese Spring wheat. This was the first comprehensive study of the MYB family in the Triticeae. The expression analysis showed that 16 genes were involved in the response to salinity, 16 genes were involved in the response to polyethylene glycol (PEG), and 11 genes were involved in resistance to low temperatures [32]. Our analyses suggest that members of the MYB superfamily are specifically expressed in different tissues and at different developmental stages in wheat. Our results showed that, in the MYB superfamily, 116 tissue-specific TFs were abundantly expressed in the seed (24 members), root (34 members), leaf (21 members), and flower (31 members), while fewer MYB TFs were expressed in the stem (6 members) and crown (0 members) (Table 3). Additionally, 130 developmental stage-specific TFs of the MYB superfamily were more abundantly expressed at the seedling (47 members), vegetative (33 members), and reproductive (26 members) stages than at the ripening (14 members), dormant seed (10 members), and germinating seed (0 members) stages (Table 2).

Our data also showed that the ERF family, which contains 217 TFs, is the second largest TF family in wheat. The ERF family is a subfamily of the APETALA2 (AP2)/ERF family, which plays crucial roles in the ethylene signaling pathways and in a variety of developmental processes. Over-expression of members of the ERF family has been shown to increase plant resistance to certain pathogens and abiotic stresses. The TaERF3 TF was identified in wheat leaves at the seedling stage. This TF was shown to be involved in the early stages of the defense response *Blumeria graminis* via salicylic acid (SA) signaling, and in the later stages of the defense response to *Fusarium graminearum* and *Rhizoctonia cerealis* via ethylene/jasmonic acid signaling pathways [33]. TaPIE1-overexpressing transgenic wheat showed significantly enhanced resistance to both *R. cerealis* and freezing stress, as a result of activation of defense- and stress-related genes downstream of the ethylene signaling pathway, and altered physiology [34]. In this study, the ERF family members showed some tissue- and developmental stage-specificity. There were 84 tissue-specific ERF sequences expressed abundantly in the flower (23 members), leaf (12 members), root (24 members), and seed (23 members), but fewer ERF sequences in the stem (1 member) and crown (1 member) (Table 3). These distributions are similar to those reported by Zhuang *et al.* [35]. There were 130 developmental stage-specific TFs in the ERF family that were abundantly expressed at the vegetative (44 TFs), seedling (35 TFs), and reproductive (26 TFs) stages, but fewer expressed at the dormant seed (8 TFs) and germinating seed (0 TFs) stages (Table 2).

The third largest TF family in wheat is the NAC family. In wheat, members of the NAC family are involved in the defense response against the stripe rust pathogen and abiotic stresses. TaNAC4 in the NAC family was shown to be induced via infection with the strip rust pathogen, methyl jasmonate, abscisic acid, ethylene, and some environmental stimuli (high salinity, wounding, and low-temperature) [36]. In another study, the TaNAC8 transcript in leaves was induced by infection with the stripe rust pathogen and by methyl jasmonate and ethylene [37]. Members of the NAC family also show some tissue- and developmental stage-specificity. In wheat seedlings, the expression of TaNAC4 was higher in roots than in leaves and stems [36]. TaNAC8 was strongly expressed in developing wheat seeds, but weakly expressed in the stems and flowers [37]. Our analyses showed that NAC family members exhibit tissue- and developmental stage-specificity in wheat. In this study, 105 developmental stage-specific members of the NAC family were most abundantly expressed at the vegetative (40 TFs), seedling (33 TFs), reproductive (16 TFs), and ripening (15 TFs) stages, while few NAC family members were expressed at the dormant seed (1 TFs) and germinating seed (0 TFs) stages (Table 2). Also, 89 tissue-specific TF members of the NAC family were abundantly expressed in the seed (27 members), leaf (29 members), and flower (15 members), but few NAC family members were expressed in the stem (2 members) and crown (1 member) (Table 3). These expression patterns are similar to those reported for TaNAC4 and TaNAC8. Understanding the biological function and distribution information of these specific TFs at the family level can provide useful information for future agricultural improvements.

## Conclusion

Approximately 2.22% of the genes (2407 genes) in the wheat genome were identified as TFs and were clustered into 63 TF families. We have constructed a new wheat TF database, WheatTFDB, which integrates individual TF annotations with information on domain architecture, protein features, GO terms, developmental stage and tissue information, and genomic sequences. This represents an updated comprehensive database of wheat TFs, and includes genomic sequences, and information about developmental stages and tissues. Based on the developmental and tissue information in WheatTFDB, we identified 1257 developmental stage-specific TFs and 1104 tissue-specific TFs. Analyses at the family level revealed that the MYB superfamily and the NAC and ERF families are the three largest groups of wheat TFs. Members of these groups were widely expressed in different tissues and at different developmental stages. These data will be useful for identifying target TFs involved in the stress response at a particular developmental stage, and will be useful for functional genomic studies on wheat TFs aimed at understanding the regulatory mechanisms underlying stress responses.

## Methods

### Sequence retrieval

The expressed sequence tag (EST) and tentative consensus contigs (TC) sequences of wheat were obtained from the Gene Index Database (wheat release 12.0, ftp://occams.dfci.harvard.edu/pub/bio/tgi/data/Triticum_aestivum/). The sequences were sorted into seven different developmental stages: dormant seed, germinating seed, seedling, vegetative, reproductive, ripening, and “unclear” (integrating the sequences assigned to “unknown developmental stage”, “mixed”, and “not yet classified”). These groupings were based on information in the original EST library at the National Center for Biotechnology Information (NCBI) Unigene database (Additional file 10: Table S1, http://www.ncbi.nlm.nih.gov/unigene/). The sequences were also grouped into categories based on tissues: crown, flower (containing the sequences from the inflorescence), leaf (including sequences from the sheath), root, seed, stem, and other tissues (sequences from the callus, cell culture, whole plant, mixed tissue, not yet classified tissue, and unspecified tissue). (Additional file 10: Table S1) shows details of the classifications from the 393 wheat sequence libraries. In total, 1940 and 7112 potential TF sequences of wheat were downloaded from wDBTF (http://wwwappli.nantes.inra.fr:8180/wDBFT/) and PlantTFDB (http://planttfdb.cbi.pku.edu.cn/), respectively.

### Transcription factor prediction and annotation

The downloaded EST and TC sequences contained some redundant sequences. We used the cdhit-est program in the cdhit package to filter out the redundant sequences in the downloaded wheat data (−c 0.95 –n 8) [38], and obtained a set of 235,978 non-redundant sequences. The nucleic acid sequences were translated into proteins using the framefinder program in the ESTate package (Expressed Sequence Tag Analysis Tools Etc., http://www.ebi.ac.uk/~guy/estate/) [39]. Then, we used the cd-hit clustering program to generate a set of 174,867 non-redundant proteins (−c 0.95 –n 5).

Transcription factors contain conserved sequence regions, DBDs, which define them as a TF. The TFs can be grouped into families based on their DBDs [12,13]. The 69 TF families in this research were identified based on the family assignment rules described in PlantTFDB V3.0 (http://planttfdb.cbi.edu.cn/) [14] and PlnTFDB V3.0 [15]. Details are shown in Additional file 11: Note S1. The HMMER V3.0 package (http://hmmer.janelia.org/) was used to build hidden Markov model (HMM) profiles to identify the wheat TFs. In this study, multiple sequence alignment seeds of 59 TF families were acquired in the Pfam database (http://pfam.sanger.ac.uk/) for the HMMER search. There were 10 TF families without available multiple sequence alignment seeds in the Pfam database, and so we downloaded their seeds from the PlnTFDB. Next, we constructed the HMM profiles of the 69 TF families from their multiple sequence alignment seeds using the hmmbuild program in HMMER V3.0. These HMM profiles were used to identify TFs. The hmm search program in the HMMER V3.0 package was used to predict TFs by searching the non-redundant wheat proteins with each HMM profile. The protein sequences matching the HMM profiles (e-value < 0.01) were considered to be TFs. Some TFs identified in this phase were distributed into more than one family. We detected and removed redundant TF sequences as described by Iida [11]. The non-redundant TFs in the 69 families are shown in Table 1. Additional details are listed in Additional file 1: Table S2, and the TF sequences are listed in Additional file 12: Note S2.

The putative TFs are listed with their original developmental stage and tissue-source information in Additional file 2: Table S3 and Additional file 6: Table S4. Grünbaum’s rotationally symmetric Venn diagram with seven regions was used to show the number of TFs identified at different developmental stages and in different tissues [21] (Figures 1, 2). Some TFs were transcribed at more than one developmental stage and/or in more than one type of tissue—these were recorded as unspecific TFs. The TFs that were only expressed at one developmental stage or in one tissue were designated as specific TFs. We identified the specific TFs at different developmental stages or in different tissues in wheat (Tables 2 and 3). Specific TF identities (IDs) are listed in Additional file 1: Table S2. The developmental- and tissue-specific expression patterns of these specific TFs were validated by BLAST searches in the NCBI wheat EST database. The input sequences were the original EST sequences of the specific TFs. The BLAST search parameters were as follows: max target sequences (10), expected threshold (10), word size (28), and match/mismatch scores (1, −2). The BLAST results for the developmental stage- and tissue-specific TFs are listed in Additional file 3: Table S5 and Additional file 7: Table S6, respectively. These tables contain information about the query and subject sequences, including the identity, alignment length, query sequence, and subject sequence length. The subject sequences listed in Additional file 3: Table S5 and Additional file 7: Table S6 include matched sequences that met the following criteria: (i) the alignment length divided by the query sequence length was greater than 0.95, (ii) the identity value was no less than 0.80, and (iii) the subject sequence was longer than its query sequence.

The developmental stage-matched sequences are listed in Additional file 4: Table S7. This table shows information about the developmental stage-matched sequences, including the original developmental stage, cultivar, mRNA sequence, and BLAST results. When the developmental stage of a matched sequence in Additional file 4: Table S7 differed from its query sequence, we added the developmental stage information for the matched sequences to its query sequence in Additional file 2: Table S3 (highlighted with a red background). The tissue-matched sequences are listed in Additional file 8: Table S8. This table shows information on the matched tissue sequences including tissue type, sequence length, mRNA sequence, and BLAST results. When the tissue type of the matched sequence differed from its query sequence, the tissue type of the matched sequence was added to its query sequence in Additional file 6: Table S4 (highlighted with a red background).

We used the cdhit program to filter out a large number of redundant sequences, including EST, TC, and protein sequences. Some redundant sequences from different developmental stages and tissues had similar identities and lengths as those of non-redundant sequences, suggesting that they might be TFs. Therefore, the developmental stage and tissue information of the filtered TF sequences was also considered. Each redundant nucleic acid sequence was finally clustered and matched to a non-redundant sequence. First, we obtained the filtered-out sequences through the ID in the cdhit results. Then, we used HMMER V3.0 to identify TFs among the filtered-out sequences. According to the corresponding information between the 2407 TFs and the redundant sequences, the newly discovered developmental stage and tissue information for the filtered-out TF sequences was added to Additional file 2: Table S3 and Additional file 6: Table S4, respectively (highlighted with a green background). Based on the tissue or developmental stage information from the BLAST results and filtered-out sequences, we validated and updated the tissue type and developmental stage information in Tables 2 and 3.

To evaluate the redundancies and similarities among the three wheat TF databases (PlantTFDB, wDBTF, and WheatTFDB), we applied the cdhit program in the cdhit package at four similarity thresholds: 0.85, 0.90, 0.95, and 1.00 (Table 4).

We analyzed the percentages of TFs in the genomes of various cereals (rice, maize, wheat, Einkorn wheat, *Aegilops tauschii*, barley, *Brachypodium*, and sorghum), using genomic information published in PlnTFDB V3.0 and PlantTFDB V3.0. In PlnTFDB V3.0, the percentages of TFs in cereal genomes were calculated by dividing the number of TFs by the total number of proteins [15]. In PlantTFDB V3.0, the percentages of TFs in cereal genomes were calculated by dividing the total number of TFs by the total number of genes [14]. There are 108,569 protein-coding genes in wheat, according to the MIPS (Munich Information Center for Protein Sequences, http://plants.ensembl.org/Triticum_aestivum/Info/Annotation/#genebuild). We used the PlantTFDB V3.0 method to calculate the percentages of TFs in the wheat genome as listed at wDBTF and WheatTFDB. The computed percentages of TFs in the wheat genome are listed in Table 5.

To provide further comprehensive functional information on the individual TFs in our new database, WheatTFDB, we used InterProScan V5.3-46.0 [40] to search for protein domain identifications and GO term assignments in the signature database Panther 8.1. We also collected genomic information for these TFs. We aligned 2407 TC or EST sequences against the chromosome-based draft sequence of the wheat genome [3] by BLAT (−minIdentity 0.95) [41], but found that the search results were locally aligned and unordered. Therefore, we used DNAMAN V6.0 (Lynnon BioSoft) to integrate and validate the alignment information between the ESTs and genome sequences. The alignment information is shown in Additional file 9: Table S9.

### Availability of supporting data

All the supporting data are included as additional files.

## Additional files

Additional file 1: Table S2. The list of TF IDs and specific IDs appear in different tissues and developmental stages. Additional file 2: Table S3. TFs with their original developmental stage information. The number in the ninth column is the sum of each TF appears at the dormant seed, germinating seed, reproductive, ripening, seedling, and vegetative stages. Additional file 3: Table S5. Validation the developmental stage information of the 1326 putative specific TFs by BLAST. Additional file 4: Table S7. The list of developmental stage matched sequences. Additional file 5: Figure S1. Interpretation of Grünbaum’s 7–set Venn diagram. In Figure 1, a–g represent (a) dormant seed, (b) germinating seed, (c) reproductively, (d) ripening, (e) seedling, (f) vegetative, and (g) unclear developmental stage, respectively. In Figure 2, a–g represent (a) Crown, (b) flower, (c) leaf, (d) root, (e) seed, (f) stem and (g) other tissue, respectively. Additional file 6: Table S4. TFs with their original tissue information. Additional file 7: Table S6. Validation the tissue information of the 1233 putative specific TFs by BLAST. Additional file 8: Table S8. The list of tissue-matched sequences. Additional file 9: Table S9. The genomic information of 2407 TFs. Additional file 10: Table S1. Details of the original EST libraries from the NCBI Unigene site. Additional file 11: Note S1. Family assignment rules. Additional file 12: Note S2. TF sequences identified in this study.
