# Supplementary figures and images for: Genome-wide characterization of developmental stage- and tissue-specific transcription factors in wheat
Source: BMC Genomics. 2015 Feb 25;16(1):125. doi: 10.1186/s12864-015-1313-y (PMC4344791; doi:10.1186/s12864-015-1313-y)

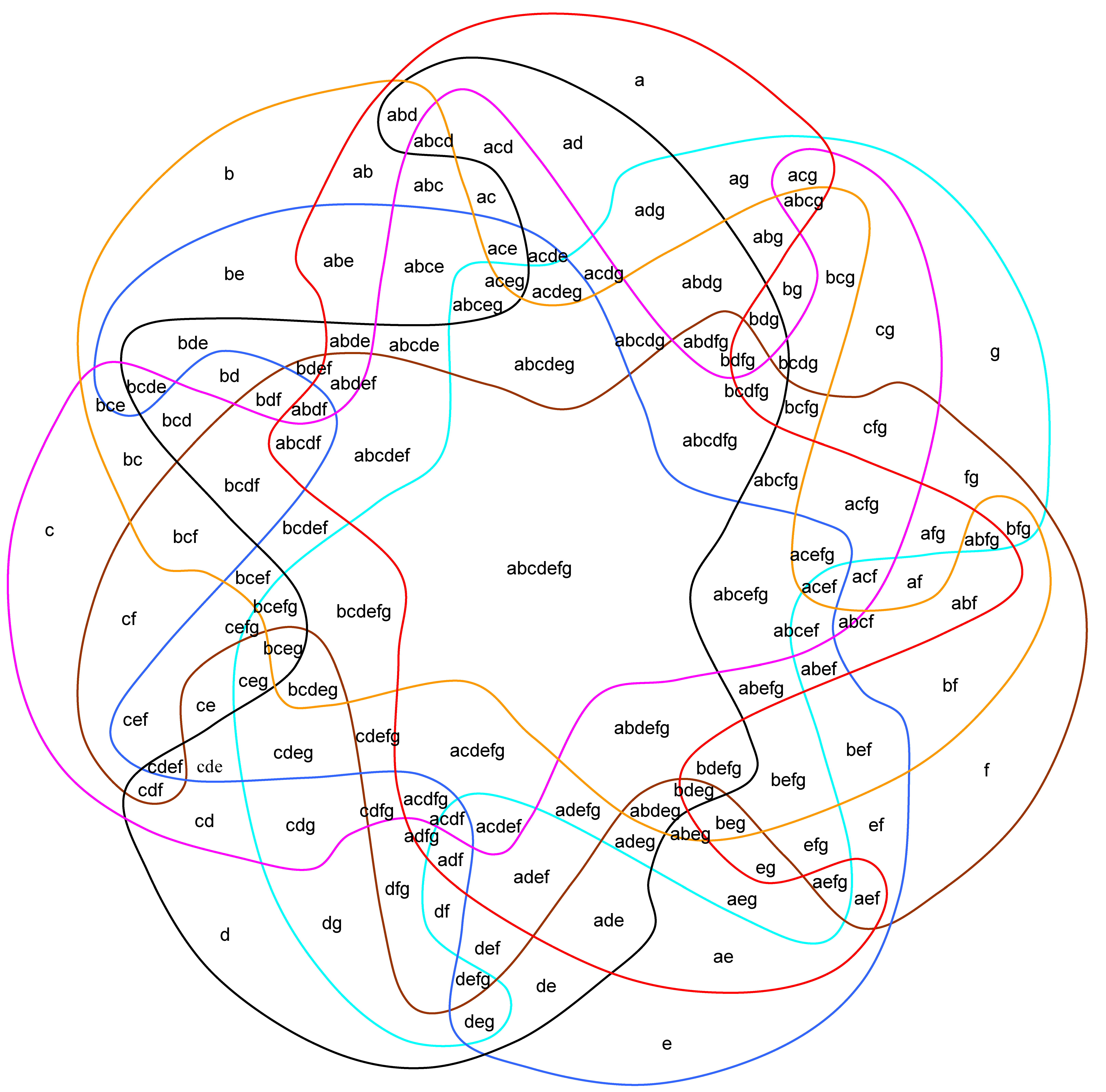

Supplement: Additional file 5: Figure S1. — Interpretation of Grünbaum’s 7–set Venn diagram. In Figure 1, a–g represent (a) dormant seed, (b) germinating seed, (c) reproductively, (d) ripening, (e) seedling, (f) vegetative, and (g) unclear developmental stage, respectively. In Figure 2, a–g represent (a) Crown, (b) flower, (c) leaf, (d) root, (e) seed, (f) stem and (g) other tissue, respectively. [file 12864_2015_1313_MOESM5_ESM.jpeg]
