# Supplementary material for: Genome-wide characterization of developmental stage- and tissue-specific transcription factors in wheat
Source: BMC Genomics. 2015 Feb 25;16(1):125. doi: 10.1186/s12864-015-1313-y (PMC4344791; doi:10.1186/s12864-015-1313-y)
Supplement: Additional file 11: Note S1. — Family assignment rules. [file 12864_2015_1313_MOESM11_ESM.doc]

| **Family** | | DNA-binding domain | Auxiliary domain | Forbidden domain |
| --- | --- | --- | --- | --- |
| AP2/ERF | AP2 | AP2 (>=2) (PF00847) |  |  |
|  | ERF | AP2 (1) (PF00847) |  |  |
|  | RAV | AP2 (PF00847)  B3 (PF02362) |  |  |
| B3 superfamily | ARF | B3 (PF02362) | Auxin_resp (PF06507) |  |
|  | B3 | B3 (PF02362) |  |  |
| BBR-BPC | | GAGA_bind (PF06217) |  |  |
| BES1 | | DUF822 (PF05687) |  |  |
| bHLH | | HLH (PF00010) |  |  |
| bZIP | | bZIP_1 (PF00170) |  |  |
| C2C2 | CO-like | zf-B_box (PF00643) | CCT (PF06203) |  |
|  | Dof | zf-Dof (PF02701) |  |  |
|  | GATA | GATA(PF00320) |  |  |
|  | LSD | zf-LSD1 (PF06943) |  | Peptidase_C14 (PF00656) |
|  | YABBY | YABBY (PF04690) |  |  |
| C2H2 | | zf-C2H2 (PF00096) |  | RNase_T (PF00929) |
| C3H | | zf-CCCH (PF00642) |  | RRM_1 (PF00076)  or Helicase_C (PF00271) |
| CAMTA | | CG-1 (PF03859) |  |  |
| CPP | | TCR (PF03638) |  |  |
| DBB | | zf-B_box (>=2) (PF00643) |  |  |
| E2F/DP | | E2F_TDP (PF02319) |  |  |
| EIL | | EIN3 (PF04873) |  |  |
| FAR1 | | FAR1 (PF03101) |  |  |
| GARP | ARR-B | G2-like (Download from PlnTFDB) | Response_reg (PF00072) |  |
|  | G2-like | G2-like (Download from PlnTFDB) |  |  |
| GeBP | | DUF573 (PF04504) |  |  |
| GRAS | | GRAS (PF03514) |  |  |
| GRF | | WRC (PF08879) | QLQ (PF08880) |  |
| HB | HD-ZIP | Homeobox (PF00046) | HD-ZIP_I/II  or START (PF01852) |  |
|  | TALE | Homeobox (PF00046) | BELL  or ELK (PF03789) |  |
|  | HB-PHD | Homeobox (PF00046) | PHD (PF00628) |  |
|  | HB-other | Homeobox (PF00046) |  |  |
| HRT-like | | HRT-like (Download from PlnTFDB) |  |  |
| HSF | | HSF_dna_bind (PF00447) |  |  |
| LBD (AS2/LOB) | | DUF260 (PF03195) |  |  |
| LFY | | FLO_LFY (PF01698) |  |  |
| MADS | M-type | SRF-TF (PF00319) |  |  |
|  | MIKC | SRF-TF (PF00319) | K-box (PF01486) |  |
| MYB superfamily | MYB | Myb_dna_bind (>=2) (PF00249) |  | SWIRM  (PF04433) |
|  | MYB_related | Myb_dna_bind (1) (PF00249) |  | SWIRM  (PF04433) |
| NAC | | NAM (PF02365) |  |  |
| NF-X1 | | Zf-NF-X1 (PF01422) |  |  |
| NF-Y | NF-YA | CBFB_NFYA (PF02045) |  |  |
|  | NF-YB | NF-YB (Download from PlnTFDB) |  |  |
|  | NF-YC | NF-YC (Download from PlnTFDB) |  |  |
| Nin-like | | RWP-RK (PF02042) |  |  |
| NZZ/SPL | | NOZZLE (PF08744) |  |  |
| S1Fa-like | | S1FA (PF04689) |  |  |
| SBP | | SBP (PF03110) |  |  |
| SRS | | DUF702 (PF05142) |  |  |
| TCP | | TCP (PF03634) |  |  |
| Trihelix | | Trihelix (Download from PlnTFDB) |  |  |
| VOZ | | VOZ (Download from PlnTFDB) |  |  |
| Whirly | | Whirly (PF08536) |  |  |
| WRKY | | WRKY (PF03106) |  |  |
| ZF-HD | | ZF-HD_dimer (PF04770) |  |  |
| FHA | | FHA (PF00498) |  |  |
| LIM | | LIM(PF00412) |  |  |
| PLATZ | | PLATZ (PF04640) |  |  |
| TUB | | Tub (PF01167) |  |  |
| ULT | | ULT (Download from PlnTFDB) |  |  |
| TAZ | | zf-TAZ(PF02135) |  | PHD (PF00628) |
| Alfin-like | | Alfin-like (Download from PlnTFDB) |  |  |
| BSD | | BSD (PF03909) |  |  |
| CSD | | CSD (PF00313) |  |  |
| DBP | | DNC (Download from PlnTFDB)  PP2C (PF00481) |  |  |
| OFP | | Ovate(PF04844) |  |  |
| Sigma70-like | | sigma70_r2 (PF04542)  sigma70_r3 (PF04539)  sigma70_r4 (PF04545) |  |  |
| Tify | | tify (PF06200) |  | GATA(PF00320) |
| mTERF | | mTERF (PF02536) |  |  |
